# Evaluating the Knowledge and Information-Seeking Behaviors of People Living With Multiple Sclerosis: Cross-Sectional Questionnaire Study

**DOI:** 10.2196/63763

**Published:** 2025-02-25

**Authors:** Véronique Duguay, Dominique Comeau, Tiffany Turgeon, Nadia Bouhamdani, Mathieu Belanger, Lyle Weston, Tammy Johnson, Nicole Manzer, Melissa Giberson, Ludivine Chamard-Witkowski

**Affiliations:** 1 Centre de Formation Médicale du Nouveau-Brunswick Université de Sherbrooke Moncton, NB Canada; 2 Vitalité Health Network Dr. Georges-L.-Dumont University Hospital Centre Moncton, NB Canada; 3 Department of Chemistry and Biochemistry Faculty of Science Université de Moncton Moncton, NB Canada; 4 Vitalité Health Network Medical Genetics Department Dr Georges-L.-Dumont University Hospital Center Moncton, NB Canada; 5 Department of Neurology The Moncton City Hospital Moncton, NB Canada; 6 Multiple Sclerosis Clinic The Moncton City Hospital Moncton, NB Canada; 7 Department of Neurology Dr. Georges-L.-Dumont University Hospital Center Moncton, NB Canada

**Keywords:** multiple sclerosis, chronic illness, misinformation, web-based searches, education, health information, social media, health literacy, patient-doctor relationship, health-related information, information-seeking behavior

## Abstract

**Background:**

The internet has emerged as a primary source of health-related information for people living with multiple sclerosis (MS). However, given the abundance of misinformation found on the web, this behavior may pose a significant threat to internet users.

**Objective:**

This study aims to explore the knowledge and information-seeking behavior of people living with MS followed at a specialized MS clinic where education is a cornerstone of care.

**Methods:**

This cross-sectional survey–based study comprised 20 true or false statements, covering both scientific facts and popular misinformation about MS treatments. A “scientific fact score” and a “misinformation score” were calculated by attributing a scoring system to each point in the survey: +1 point was attributed to correct answers, –1 point was attributed to incorrect answers, and 0 point was attributed to “I don’t know.” Furthermore, the survey inquired about participants’ health-seeking behaviors.

**Results:**

The mean age of the 69 participants was 48.4 (SD 10.9) years, 78% (54/69) were female, 81% (56/69) were highly educated, 90% (62/69) were receiving a disease-modifying therapy, and 52% (30/58) had experimented with alternative therapies. The mean score for answering the scientific and misinformation questions correctly was 69% (SD 2.4%) and 22% (SD 4.5%), respectively (*P*<.001). Notably, when questioned about misinformation, answering correctly dropped significantly (*P*<.001), while indecision (*P*<.001) and answering incorrectly (*P*=.02) increased. Sociodemographic factors and medical questions were not significantly associated with scientific and misinformation scores (all *P*>.05); however, misinformation scores did significantly correlate with levels of education (*P*=.04). The main sources of health-related information were from expert-led MS websites (48/58, 82%) and health care professionals (34/58, 59%). Low-reliability sources were less used; however, word of mouth seemed to be prevalent (14/58, 24%), followed by Facebook (10/58, 17%). On average, people with MS reported having consulted 3 high- to moderate-quality sources and only 1 low-quality source.

**Conclusions:**

Education at the clinic and consulting primarily moderate- to high-quality sources did not safeguard against misinformation, indicating a need for more misinformation-geared education at the clinic. Notably, there is a need to proactively educate patients about misinformation commonly found on the web, and more importantly, create space for them to discuss the information without prejudice. As novel educational methods may be relatively more time-consuming, implementing change may be challenging. Furthermore, age, sex, education level, and health literacy might not safeguard against misinformation. Herein, we were unable to identify correlations associated with scores obtained on the questionnaire other than educational level. Although the educational level did seem to impact the misinformation score, this did not stop participants from experimenting with alternative therapies. Although studies are exploring novel ways to effectively deal with health misinformation on the web, more research is needed to fully understand this highly complex social phenomenon.

## Introduction

Although patients can acquire information regarding health from a multitude of sources such as health care professionals, pharmaceutical companies, books, pamphlets, radio, and television, the majority will tend to get their information on the web [1-3]. Given the large number of unreliable sources and inaccurate information currently flooding the internet, patients can easily become misinformed [3]. Misinformation can be defined as information that is not in accordance with the leading scientific consensus [4]. Although misinformation is widespread across the web-based sphere, social media sources including Twitter, YouTube, Facebook, and WhatsApp have been found to be the predominant outlets of misinformation [3,5]. With most of the health information on the web assessed as being of low-quality, inaccurate, and incomplete [6,7], users are at an increased risk of being deceived by misinformation. This is further exacerbated by the fact that false information diffuses significantly farther, faster, deeper, and more broadly than veracious information [8-10]. While web-based searches can empower patients [11] and improve their knowledge, health literacy, capacity to manage their health [12], and the patient-doctor relationship [13], misinformation may also lead individuals to erroneous conclusions and potential harm [4]. Patients may be particularly vulnerable to hopes of recovery driven by misinformation, myths, and unproven therapies [4].

For those living with multiple sclerosis (MS), the internet is becoming an important source of health-related information as well as a source of community support through the exchange of lived experience [14-19]. For people with MS, information found on the internet may help in coping with the disease and provide some reassurance in their chosen therapeutic modality [15]. More than half of people with MS used mass media sources rather than interpersonal information sources such as health care providers and advocacy organizations, among others [14]. Thus, addressing the reliability of the information found on the internet is a critical issue [18] as people with MS are likely to be affected by misinformation [20,21]. Notably, a recent study evaluating the quality of YouTube videos regarding MS found that more than half lacked high-quality content [20]. Nevertheless, studies have indicated that people with MS still believe the most trusted information source to be their physician [14,18], and a large proportion remain skeptical about the quality of the information they gather on the web [14,17], with 40% stating they had concerns [14].

With both web-based information and the global population with MS on the rise [22], a growing number of people with MS are exposed to potential harm through unverified therapies, potentially impacting their security and health [23]. Indeed, most people with MS will use complementary and alternative medicine seen on various social media platforms [24-26]. Although seemingly well tolerated, these unproven therapies may interact adversely with medical treatments, pose harm, and incur financial costs [23,25]. The purpose of this study was to evaluate if people with MS at a specialized MS clinic were able to correctly answer questions about their illness as well as identify misinformation about alternative therapies. Other objectives were to identify where people with MS get their health-related information and identify correlations associated with questionnaire scores.

## Methods

### Research Design

People living with MS and followed at the MS clinic in Moncton, New Brunswick, Canada, were invited to take part in the survey-based study between April and July 2023. More specifically, participants who agreed to be contacted by the research team were telephoned and informed of the study. To be included, people with MS had to be aged 19 years or older to provide informed consent.

### Ethical Considerations

The study was approved by the Vitalité Health Network Research Ethics Board (101729). If the participant decided to participate, they were asked to sign the consent form after which they were invited to fill out the survey questionnaire. Data was de-identified to ensure privacy and confidentiality. Participants were not compensated.

### Participant Demographics

Participants included 69 people living with MS followed at a specialized MS clinic providing management, education, and treatment of MS. More specifically, 90 potential participants were contacted for the study and 69 were accepted: giving a participation rate of 77%. At the clinic, people living with MS are followed by a clinical team comprised of 1 neurologist and 3 nurses as well as neurologists on standby. They have access to the clinical team 5 days per week, which they can readily contact by telephone or by email. Initial consultations are comprised of a thorough educational session that relays information about the disease such as physiopathology, symptoms, disease evolution, as well as medication. During this session, they are given resources to consult for disease-related information; they are also encouraged to contact their clinical team if they have any questions and to be wary of nonexpert sources. Subsequent visits are supplemented with information on relevant symptoms, disease evolution, and medication tailored to their specific needs.

### Survey Instrument and Data Collection Procedure

A 20-item “true or false” survey was developed by neurologists and nurses, which comprised 10 scientific facts and 10 popular misinformation statements regarding MS (Multimedia Appendix 1). Scientific facts and misinformation statements were distributed at random in the questionnaire. For each item, participants were asked to respond if they considered the statement to be true, partially true, do not know, partially false, or false. The 10 scientific facts consisted of accurate scientific truths about MS, while the 10 misinformation statements consisted of erroneous statements about the efficacy of alternative medicine in the treatment of MS. The latter was developed based on purportedly effective alternative medicine options found on the web and on social media. Hence, the correct answers for this survey were true for all scientific facts and false for misinformation statements. A “scientific fact score” and a “misinformation score” were calculated by attributing a scoring system to each point in the survey: +1 point was attributed to correct answers (true or partially true for facts and false or partially false for misinformation statements), –1 point was attributed to incorrect answers, and 0 point was attributed to “I don’t know.” A range of –10 to 10 points could therefore be obtained for each of the scientific fact score and misinformation score. The survey also collected sociodemographic information, the participants’ use of disease-modifying therapies and alternative medicine, and the sources used to get health-related information. The sources used to get health-related information were classified as either high to moderate reliability or low reliability. The classification was done by the neurologist and nurses at the clinic and was based on source expertise (ie, the ability of the source to give accurate information) and trustworthiness (ie, the willingness of the source to provide accurate information). Education was classified as either “high” or “low”; high education included all postsecondary education diplomas (ie, university and college diplomas) and low education included high school–level diplomas or less.

### Statistical Analysis

ANOVA post hoc Sidak was used to compare means between multiple groups and the nonparametric Mann-Whitney *U* test was used to test between 2 samples. Multiple regression analysis was used to analyze the relationship between scores and several independent variables. All statistical tests were performed using GraphPad Prism (version 10.1.0; GraphPad Software Inc). Parametric tests were used after data passed normality tests (D’Agostino-Pearson omnibus, Anderson-Darling, Shapiro-Wilk, and Kolmogorov-Smirnov).

## Results

### Overview

The mean age of the 69 participants was 48.4 (SD 10.9) years and the mean time of MS diagnosis for participants was 10.9 (SD 7.8) years (Table 1). A total of 78% (54/69) of participants identified as female, 20% (14/69) as male, and 1.4% (1/69) as nonbinary (Table 1). A total of 81% (54/69) of participants were classified as highly educated (Table 1). Additionally, 90% (62/69) of participants were receiving disease-modifying therapies and 52% (30/58) reported experimenting with alternative medicine (Table 1).

**Table 1:** Respondent demographics and clinical information (N=69).

Response			Values
Age (years), mean (SD)			48.4 (10.9)
**Sex, n (%)**			
	Female	54 (78)	
	Male	14 (20)	
	Nonbinary	1 (1)	
**Education, n (%)**			
	High	56 (81)	
	Low	13 (19)	
Time since MS^a^ diagnosis (years), mean (SD)			10.9 (7.8)
**Receiving disease-modifying therapy, n (%)**			
	Yes	62 (90)	
	No	7 (10)	
**Using alternative medicine^b^, n (%)**			
	Yes	30 (52)	
	No	28 (48)	

^a^MS: multiple sclerosis.

^b^Out of the total 69 patients, only 58 answered this question.

### People With MS Were More Knowledgeable About Scientific Fact Than Misinformation

**Figure 1:**
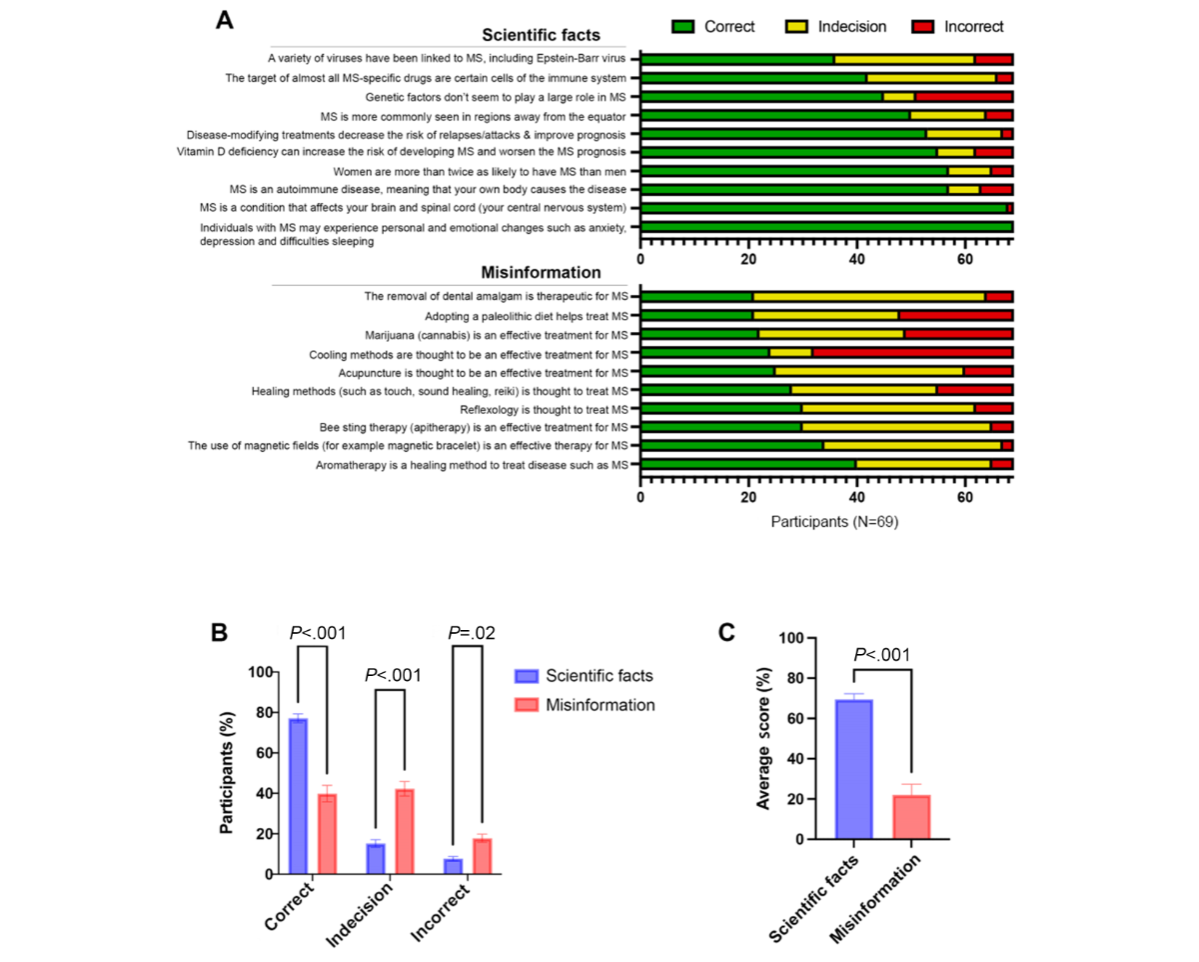
Scientific facts and misinformation identification by people with MS. (A) Answer distribution (correct, indecision, and incorrect) for scientific facts and misinformation per question. (B) Differences in answer distribution between scientific facts and misinformation (Mann-Whitney U test). (C) Average score differences between scientific facts and misinformation (ANOVA, post hoc Sidak). MS: multiple sclerosis.

Overall, when asked whether scientific facts about their disease were true or false, most people with MS were able to answer correctly (Figure 1A). Comparatively, relatively fewer participants could accurately identify misinformation. Notably, all participants correctly answered that “individuals with MS experience personal and emotional changes such as anxiety, depression, and difficulties sleeping,” and almost all participants correctly answered that “MS is a condition that affects your brain and spinal cord (your central nervous system).” More complex facts, such as “genetic factors don’t seem to play a large role in MS,” “a variety of viruses have been linked to MS, including Epstein-Barr virus,” and “the target of almost all MS-specific drugs are certain cells of the immune system,” seemed to be lesser known. The most incorrectly answered misinformation statements were that “cooling methods are thought to be an effective treatment for MS,” “adapting a paleolithic diet helps treat MS,” and that “marijuana (cannabis) is an effective treatment for MS.”

When comparing the scientific fact and misinformation score, participants were better able to correctly answer scientific facts (*P*<.001), indecision (answering “I do not know”) was more prevalent for misinformation statements (*P*<.001), and answering incorrectly significantly increased in the case of misinformation (*P*=.02; Figure 1B). Correspondingly, the average scientific fact and misinformation scores of people with MS were 69% (SD 2.4%) and 22% (SD 4.5%), respectively (Figure 1C).

### People Living With MS Used High- to Moderate-Reliability Sources for Their Disease-Related Information and Minimal Low-Reliability Sources

**Figure 2:**
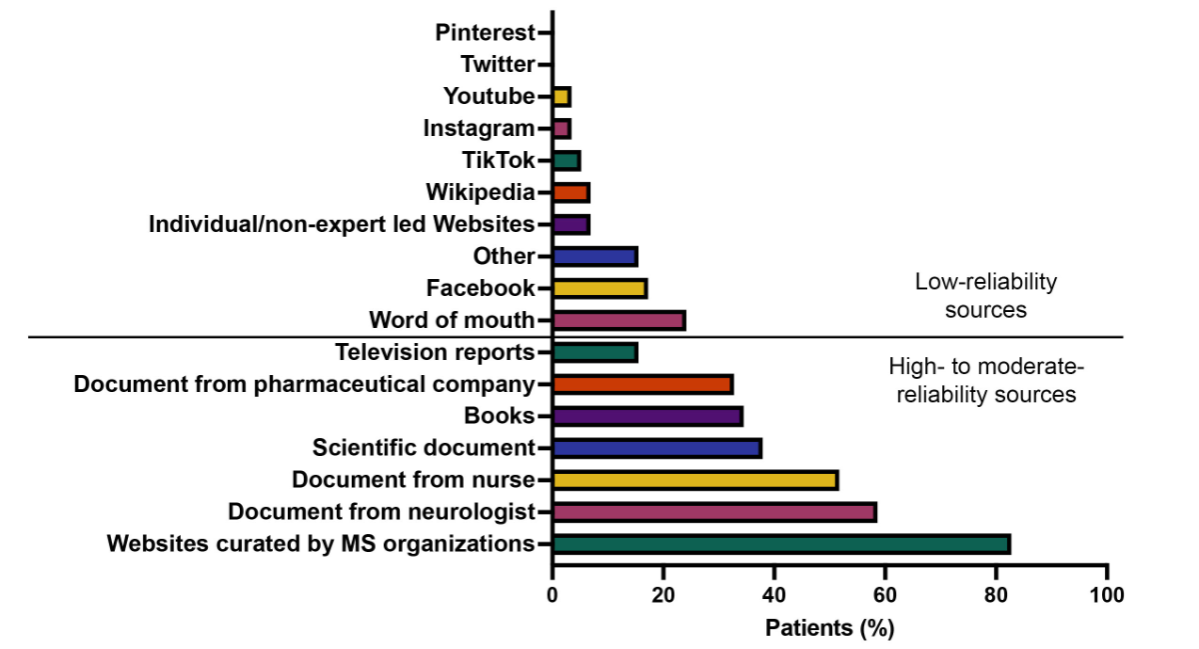
Sources used to get disease-related health information about MS. Health-related information sources are divided into 2 categories depending on their reliability: low-reliability and high- to moderate-reliability sources. MS: multiple sclerosis.

People living with MS were asked to share where they got their health-related information about their illness. The sources were classified as either high- to moderate-reliability sources or low-reliability sources (Figure 2). The great majority of participants from the specialized clinic reported getting their information from high- to moderate-reliability sources, notably, from trustworthy and expert-led MS websites (48/58, 82%) and health care professionals, namely, neurologists and nurses (34/58, 59% and 30/58, 52%, respectively). Participants also got their information from scientific documents (22/58, 38%) like peer-reviewed publications, books (20/58, 34%), and pharmaceutical companies (19/58, 33%). Low-reliability sources were less used; however, word of mouth seemed to be prevalent (14/58, 24%), followed by Facebook (10/58, 17%). On average, people with MS reported having consulted 3 high- to moderate-quality sources and only 1 low-quality source.

In linear regression analyses, we did not find that scientific score correlated with age, sex, level of education, use of disease-modifying therapies, alternative therapies, nor the use of high- to moderate- or low-reliability sources (Table 2). Misinformation score also did not correlate with age, sex, use of disease-modifying therapies, alternative therapies, or the use of high- to moderate- or low-reliability sources (Table 3). However, we observed that lower levels of education were associated with significantly fewer correct answers in the misinformation score (*P*=.04).

**Table 2:** Linear regression between various factors and MS^a^ scientific fact score (N=69).

Factors	β (95% CI)	*P* value^b^
Age	–0.3662 (–0.9019 to 0.1695)	.18
Sex^c^	0.037 (–12.23 to 16.52)	.77
Education	–4.093 (–20.26 to 12.08)	.61
Disease-modifying therapy	3.31 (–20.39 to 27.01)	.78
Alternative therapy	6.209 (–5.772 to 18.19)	.30
High to moderate-reliability sources	3.589 (–0.5315 to 7.709)	.09
Low-reliability sources	–0.9313 (–7.487 to 5.624)	.78

^a^MS: multiple sclerosis.

^b^*P* value statistically significant when <.05.

^c^A participant identifying as nonbinary was excluded from the analysis.

**Table 3:** Linear regression between various factors and MS^a^ misinformation score (N=69).

Factors	β (95% CI)	*P* value^b^
Age	–0.1946 (–1.180 to 0.7907)	.69
Sex^c^	0.094 (–37.25 to 16.57)	.45
Education	–30.85 (–60.59 to –1.108)	.04
Disease-modifying therapy	19.07 (–31.77 to 69.91)	.46
Alternative therapy	–4.905 (–30.61 to 20.80)	.70
High to moderate-reliability sources	–0.6062 (–9.445 to 8.233)	.89
Low-reliability sources	0.2161 (–13.85 to 14.28)	.97

^a^MS: multiple sclerosis.

^b^*P* value statistically significant when <.05.

^c^A participant identifying as nonbinary was excluded from the analysis.

## Discussion

### Principal Findings

We investigated the knowledge and information-seeking behaviors of people living with MS at a specialized MS clinic. Taken together, participants generally correctly identified scientific facts as being true but had greater difficulties identifying misinformation about alternative therapies as false.

Given that participants were followed by a specialized clinic, where education is a cornerstone of care, the high scoring associated with scientific facts was expected. However, even when equipped with education and a team of experts to answer questions and concerns, participants still scored relatively poorly when faced with misinformation. This may indicate a need for more misinformation-geared education but also a certain wariness of health care on the part of participants. Indeed, it was previously reported that although 82% of people with MS gathered information on the web, only 36% discussed this information with their physician [16]. It was proposed that wariness of health care may lead to patients opting out of communicating and discussing information found on the web with health care professionals, which in turn may lead to therapeutic nonadherence. Overall, this may indicate the need to proactively educate patients about misinformation commonly found on the web, and more importantly, talk openly with patients about misinformation in hopes of creating space for them to discuss information found on the web without prejudice. Indeed, people presented with accessible evidence-based corrections can reduce their belief in misinformation [4]. Although participants scored lower on the portion of the questionnaire dealing with misinformation, they more often chose to answer “I do not know.” This cautiousness was also previously documented in people with MS surveyed about web-based searches [14,17]. This healthy skepticism could represent a facilitator when educators are tasked with discrediting misinformation.

In addition, patients often will have difficulty understanding and remembering information communicated by their health care provider [27-29]. Unfortunately, understanding complex health information and applying it to everyday life, that is, health literacy, is pivotal to self-management and improved health outcomes [30,31]. Gaps in understanding may be explained by the overuse of medical terminology while gaps in recall may be due to inadequate educational methods and interventions used by health care professionals [32-34]. Therefore, compounding the issue of misinformation are the challenges that come with educating patients. It has been shown that health care professionals tend to overestimate their own ability to communicate clearly with patients [27-29,35]; thus, being aware of such gaps in communication and implementing new strategies could ameliorate patient understanding of misinformation. There are also many barriers and issues in access to health care. One commonly identified barrier for neurologists in the care of people living with MS is time constraints and availability of appointments [36]. As educational methods may be relatively more time-consuming, implementing change may be challenging [37].

Health literacy is the ability to seek, find, and understand health information on the web to help support decision-making about one’s health [4]. Low health literacy may increase a patient’s susceptibility to misinformation; however, the great majority of people consult low-quality websites for health-related information regardless of their health literacy level [4]. This could be explained by the fact that low-quality sources may be more easily understood or more engaging by eliciting stronger feelings [8]. Health literacy is also correlated with the individual’s educational level [4]. In this study, 81% (56/69) of participants had a high level of education (college or university level diplomas); thus, we could assume that the health literacy of participants was adequate. Furthermore, as MS disproportionally affects female over male individuals, the surveyed population was mostly comprised of the former. In this highly educated group, mostly comprised of female individuals, half had experimented with alternative therapies. In the realm of cancer, those most likely to use alternative medicine are indeed educated female individuals from higher-income households [38]. Furthermore, social media platforms have gained in popularity among health information seekers regardless of age or sex [39]. Consequently, age, sex, education level, and health literacy might not safeguard against web-based misinformation. Herein, we were unable to identify correlations associated with scores obtained on the questionnaire other than educational level. Although the educational level did seem to impact the misinformation score, this did not stop participants from experimenting with alternative therapies.

The belief that cannabis or diet interventions may be effective treatment alternatives for MS was notable in the surveyed population. This is of some concern as there is little data currently supporting the use of these avenues in the treatment of MS. Although some cannabinoid products may help reduce the severity of spasticity short term, it is still uncertain what the effects are on chronic neurological pain and health-related quality of life [40]. Furthermore, cannabinoids may cause adverse effects leading to treatment discontinuation as well as nervous system and psychiatric disorders [40]. More studies are needed to evaluate the efficacy and safety of using these products. Likewise, the effects of dietary interventions for MS-related outcomes remain uncertain [41]. Misconceptions relating to cannabis use and special diets have previously been identified as prevalent health misinformation for all individuals seeking health information on the web [5], demonstrating that widespread misinformation may affect a broad spectrum of individuals. This may indicate a need for larger-scale antipropaganda government initiatives. Many participants also believed that cooling methods are effective treatments. Although these modalities have demonstrated some effectiveness, no study has assessed comfort and adherence. Moreover, these methods are costly, not accessible, and have not been widely tested [42]**.**

Interestingly, participants in this small cohort mostly used high to moderate web-based sources for their MS-related health information; yet the majority were still subject to misinformation. Other than the lack of misinformation-geared education, this may be explained by the fact that people might not know where they pick up misinformation [4]. For instance, misinformation might cross one’s attention while casually scrolling on social media. Belief in misinformation may also be explained by the fact that people with MS will report an improvement in their fatigue and mood with the use of alternative therapies. Hence, it is plausible that study participants who have tried alternative therapies with some positive outcomes may earnestly believe in their efficacy and subsequently be susceptible to misinformation. Unfortunately, these alternative treatments are not without risk, as they may also negatively impact the effectiveness of disease-modifying therapies [25]. Furthermore, although all patients at the specialized MS clinic received disease-related documents to consult with their health care provider, only about half referred to these when searching for information about MS. This may illustrate a need to make health information distributed by health care professionals more engaging and accessible to people with MS, and this for a diverse group of people.

Science and health misinformation can be defined as information that is not in accordance with the leading scientific consensus. This process of delineating what is considered true or false is highly dynamic and is subject to change based on current evidence. MS is a complex disease with many unanswered fundamental questions relating to causation and susceptibility, and although disease-modifying treatments are available for relapsing-remitting MS, there remains a paucity of effective treatments for the progressive forms of MS [43]. Hopes of finding answers to unaddressed questions may increase susceptibility to misinformation, and more importantly, the lack of effective therapeutic avenues in advanced disease may encourage experimenting with alternative medicine. Furthermore, as new data emerges, what is presently considered alternative medicine may become common practice with new research highlighting efficiency and safety. Notably, some recent work has demonstrated that a Mediterranean diet may help alleviate some MS symptoms; however, more robust data are needed to validate these findings [44]. Also, a recent study has demonstrated that the Epstein-Barr virus is linked to MS, but not other previously reported viruses [45]. This highly dynamic process and changing landscape may be challenging not only for people with MS but also for educators. Although studies are exploring novel ways to effectively deal with health misinformation on the web, more research is needed to fully understand this highly complex social phenomenon [4].

### Study Limitations

This study is not without its limitations. Notably, the small sample size and the homogenous population may not accurately represent the whole population with MS. Indeed, with only 69 participants, extrapolation of the data to the larger population with MS would be precipitous; larger multicentric cohort studies are needed to better understand this phenomenon. Participants possessed a high level of education, a notable contrast to the 57.2% of Canadians with a similar educational background as reported in 2021, which may indicate that data may not be generalizable [46]. We also assume that education levels equate to health literacy; however, assessing health literacy may have been more informative. Further, participants in this study received education regarding their disease at a specialized MS clinic, suggesting that repeating this study with people with MS who have limited access to a neurologist or clinical team may yield even more worrisome results. Furthermore, some aspects of the questionnaire may have been difficult to understand for people outside of health care or the scientific field. More specifically, delineating the difference between disease-modifying and symptomatic treatments may have been challenging for some. For instance, marijuana and cooling may be considered potentially effective symptomatic treatments but not effective disease-modifying ones, which may have led participants to answer wrongfully. The wording in elements of the questionnaire may also have been confusing; the use of “seem” and “large” in “genetic factors don’t seem to play a large role in MS” may have been too vague and thus confusing to respondents. Such misinterpretations may be avoided by using standardized survey tools for assessing susceptibility to misinformation; however, more research is needed to develop such tools in MS. Interestingly, a parallel may also be drawn between questionnaire understandability/clarity and patient education; how do we make information understandable, mitigate the risk of misinterpretation, whilst ensuring that the content remains engaging? In addition, how can we assess if patients have accurately assimilated the content?

In conclusion, we show that people with MS followed at a specialized MS clinic can readily answer questions regarding their disease; however, they are potentially still being impacted by misinformation. As web-based information has the potential to improve the patient-doctor relationship and aid communication as well as help patients be more actively involved in decision-making, disparaging the use of the internet is not a reasonable solution to mitigate the potentially negative effects of misinformation. Rather, as a first line of defense, we propose that a portion of educational sessions be dedicated to thwarting misinformation as well as opening lines of communication between patients and physicians regarding information found on the web.

## Appendix

Multimedia Appendix 1 Participant questionnaire.

## Abbreviations

MS: multiple sclerosis

## Data Availability

The datasets generated during and/or analyzed during this study are available from the corresponding author on reasonable request.
