# Supplementary material for: Evaluating the Knowledge and Information-Seeking Behaviors of People Living With Multiple Sclerosis: Cross-Sectional Questionnaire Study
Source: J Med Internet Res. 2025 Feb 25;27:e63763. doi: 10.2196/63763 (PMC11897666; doi:10.2196/63763)
Supplement: Multimedia Appendix 1 [file jmir_v27i1e63763_app1.pdf]

Supplementary material

Misinformation: Evaluating the Knowledge and Information-Seeking Behaviours of People living with MS.

Participant Questionnaire

1. Before you start

The study has two parts.

- ***Part 1**: general questions.*
- ***Part 2**: 20 questions concerning Multiple Sclerosis (MS).*

2. Part 1: General Questions

1. How old are you?
2. What gender do you identify as?
3. What is your highest level of education?
4. How long ago (years) were you diagnosed with MS?
5. Do you receive specific treatment for MS? If yes, please indicate the treatment below.
6. Have you ever tried **alternative therapies** for MS? Alternative therapies include practices not prescribed by your neurologist such as massages, acupuncture, tai chi, nutrition, and reiki, amongst others. If yes, please list them below.
7. Where do you find information about multiple sclerosis (MS)? Please specify.

o Document from your neurologist
o Document from your nurse
o Document from pharmaceutical company
o Scientific document
o Government or official Websites curated by MS organizations (ex. MS Society)
o Television reports
o Books
o Individual/non-expert led Websites

- Wikipedia
- Instagram
- Facebook
- Twitter
- Youtube
- TikTok
- Pinterest
- Word of mouth
- Other

3. Part 2: Questions concerning Multiple Sclerosis (MS).

1. **MS is a condition that affects your brain and spinal cord (your central nervous system).**

False	Partly false	I'm not sure	Partly true	True

2. **Acupuncture (the insertion of very thin needles through your skin at strategic points on your body) is thought to be an effective treatment for MS.**

False	Partly false	I'm not sure	Partly true	True

3. **MS is more commonly seen in regions away from the equator.**

False	Partly false	I'm not sure	Partly true	True

4. **Bee sting therapy (apitherapy) which refers to the placement of bees on specific areas on the body is an effective treatment for MS.**

False	Partly false	I'm not sure	Partly true	True

5. **Being deficient in vitamin D can increase the risk of developing MS and worsen the MS prognosis.**

False	Partly false	I'm not sure	Partly true	True

6. **Adopting a paleolithic diet (primarily meat, vegetables and nuts) helps treat MS.**

False	Partly false	I'm not sure	Partly true	True

7. **Cooling methods such as staying in air-conditioned areas, drinking cold liquid and the use of specially designed cooling suits are though to be an effective treatment for MS.**

False	Partly false	I'm not sure	Partly true	True

8. **Disease-modifying treatments decrease the risk of relapses or attacks and improve the prognosis of MS.**

False	Partly false	I'm not sure	Partly true	True

9. **A variety of viruses have been linked to MS, including Epstein-Barr virus.**

False	Partly false	I'm not sure	Partly true	True

10. **Individuals with MS may experience personal and emotional changes such as anxiety, depression and difficulties sleeping.**

False | Partly false | I'm not sure | Partly true | True

11. **The target of almost all multiple sclerosis-specific drugs are certain cells of the immune system.**

False | Partly false | I'm not sure | Partly true | True

12. **Aromatherapy is a healing method that uses essential oils from plants to treat disease such as MS.**

False | Partly false | I'm not sure | Partly true | True

13. **Healing methods (such as touch, sound healing, reiki) is thought to treat MS.**

False | Partly false | I'm not sure | Partly true | True

14. **MS is an autoimmune disease, meaning that your own body causes the disease.**

False | Partly false | I'm not sure | Partly true | True

15. Reflexology (applied pressure to specific parts, or zones, of the foot) is thought to treat MS.

False — Partly false — I'm not sure — Partly true — True

16. Marijuana (cannabis) is an effective treatment for MS due to its high content in cannabinoids.

False — Partly false — I'm not sure — Partly true — True

17. Women are more than twice as likely to have MS than men.

False — Partly false — I'm not sure — Partly true — True

18. Genetic factors don't seem to play a large role in MS.

False — Partly false — I'm not sure — Partly true — True

19. The removal of dental amalgam is therapeutic for MS (due to its small amounts of mercury released into the body and damaging the nervous system).

False — Partly false — I'm not sure — Partly true — True

20. The use of magnetic fields (for example magnetic bracelet) is an effective therapy for MS.

False | Partly false | I'm not sure | Partly true | True
